# Evidence of maternal effects on temperature preference in side-blotched lizards: implications for evolutionary response to climate change

**DOI:** 10.1002/ece3.614

**Published:** 2013-05-23

**Authors:** Dhanashree A Paranjpe, Elizabeth Bastiaans, Amy Patten, Robert D Cooper, Barry Sinervo

**Affiliations:** 1 Department of Ecology and Evolutionary Biology, University of California at Santa Cruz1156 High St., Santa Cruz, California, 95064; 2 Department of Biological Sciences, Humboldt State University1 Harpst St., Arcata, California, 95521

**Keywords:** Maternal effects, side-blotched lizards, temperature preference, thermo-regulation, throat color polymorphism

## Abstract

Natural populations respond to selection pressures like increasing local temperatures in many ways, including plasticity and adaptation. To predict the response of ectotherms like lizards to local temperature increase, it is essential to estimate phenotypic variation in and determine the heritability of temperature-related traits like average field body temperature (*T*_b_) and preferred temperature (*T*_p_). We measured *T*_p_ of *Uta stansburiana* in a laboratory thermal gradient and assessed the contribution of sex, reproductive status and throat color genotype to phenotypic variation in *T*_b_ of adult lizards. Females had higher *T*_p_ than males. However, they temporarily preferred lower temperature when gravid than when nongravid. Using a nested half-sib design for genetic crosses in the laboratory, we estimated relative contributions of additive genetic variation and maternal effects to *T*_p_ of hatchlings. Our results show that maternal effects, but not additive genetic variation, influence *T*_p_ of hatchlings in *U. stansburiana*. Maternal *T*_p_ and the presence or absence of blue throat color alleles significantly influenced *T*_p_ of hatchlings. We discuss ecological and evolutionary consequences of these maternal effects in the context of rapid climate change and natural selection that we measure on progeny survival to maturity as a function of maternal *T*_p_.

## Introduction

Natural populations experiencing a selection pressure such as increasing environmental temperatures can respond in one or more of the following ways – (i) by moving to more favorable habitats, thereby changing their geographical distribution (ii) by showing plastic responses to overcome the stressful conditions (iii) by undergoing evolutionary adaptation and (iv) going extinct. (Gienapp et al. 2008; Visser 2008). The response(s) of natural populations, although variable in space and time, may depend on factors such as the strength of selection pressure at local scale, the population's potential for phenotypic plasticity, and the evolutionary potential (available additive genetic variation) (Hoffmann and Sgro 2011). If local temperatures rise, any trait that is phenotypically plastic with respect to temperature will respond on a shorter time scale than that over which evolutionary adaptation usually occurs (Visser 2008). This can happen at the level of the individual within a year or across years, through learning, or via maternal or other epigenetic effects (across generations). On the other hand, genetic variation in traits related to physiological limits and phenological timing allows for local adaptation with respect to traits under selection in the long term (Linhart and Grant 1996). The responses to thermal heterogeneity thus occur on different time scales. Each organism's response to thermal heterogeneity depends on its size, mobility and life span. In an environment that is thermally heterogeneous, the short term consequences of thermal change will depend on the diversity of strategies that organisms can use to cope with the change (Angilletta et al. 2006), while the long term out comes will depend on the phenotypic variation available and the evolutionary potential (additive genetic variation) of the population with respect to thermal traits. The relative contributions of coping mechanisms such as phenotypic plasticity and genetic variation available within the population (evolutionary potential) can be estimated through breeding experiments in laboratory and field populations (Kingsolver et al. 2007; Visser 2008).

Ectotherms such as lizards are especially vulnerable to changes in environmental temperatures, such as local warming trends in recent years (Sinervo et al. 2010) because ambient temperatures directly influence their body temperature (*T*_b_). Body temperature, in turn, influences various aspects of their physiology, behavior and fitness (Bennett 1980; Huey and Berrigan 2001; Martin and Huey 2008). Most lizards, therefore, regulate their *T*_b_ within a narrow range when they are active (Bowker 1984; Cossins and Bowler 1987). This temperature range is referred to as activity temperature range or normal activity range (Pough and Gans 1982). Whether an ectothermic animal like a lizard is able to maintain *T*_b_ within its activity temperature range, or how long it can maintain *T*_b_ within that range, can influence many aspects of the animal's life history. These aspects include, but are not limited to, growth rates (Sinervo and Adolph 1994; Autumn and DeNardo 1995; Sinervo and Dunlap 1995), physiological performance traits such as sprint speed and endurance (Angilletta et al. 2002), and reproductive patterns (Licht 1966, 1967a,b, 1973a,b).

In lizards, effective control of body temperature within the activity temperature range is achieved through a complex suite of mechanisms that range from modifying behavior and movements (behavioral thermoregulation) to color change (Gans and Pough 1982). Behavioral thermoregulation primarily involves selection of microhabitat, timing of activity, postural adjustments, huddling, and burrowing behaviors. However, studies of thermoregulatory behavior of lizards, in well-characterized thermal environments, have revealed that insufficiently heterogeneous thermal microhabitats often prevent animals from attaining their physiologically optimal *T*_b_ in nature (Grant and Dunham 1988; Gil et al. 1994). Other physical and biotic aspects of the environment, such as extreme temperatures and predation pressure, can also prevent lizards from maintaining their *T*_b_ within the activity temperature range for part of their activity cycle. Therefore, mean body temperature selected in a laboratory thermal gradient, that is, preferred or selected temperature (*T*_p_), rather than *T*_b_ measured in field-active animals, is often used in ecological analyses (Huey and Slatkin 1976; Hutchison 1979).

Mean selected temperature (*T*_p_) is traditionally measured using small thermocouples in laboratory thermal gradients (Licht 1966) where potentially confounding aspects of the physical and social environment may be standardized (Patterson and Davies 1978; Ibargüengoytía 2005). Preferred or selected temperature (*T*_p_) has been measured as average of snapshots of body temperatures at regular intervals for some species, like nocturnal geckos (25–31°C for five different species, Angilletta et al. 1999), *Anolis carolinensis* (32°C, Licht 1966), and *Uta stansburiana* (36.5°C in field, Sartorius et al. 2002). A variety of internal and external factors can potentially influence thermoregulation, such as age (Angilletta et al. 1999), sex (Mayhew 1963; Hirth and King 1969; Licht 1973a,b; Werner and Whitaker 1978; Ibargüengoytía 2005; Huey and Pianka 2007; Lailvaux and Irschick 2007), time of day (Sinervo 1990; Refinetti and Susalka 1997; Firth and Belan 1998; Angilletta et al. 1999), and season of the year (Sievert and Hutchison 1989; Firth and Belan 1998; Ellis et al. 2008). The activity temperature range is thought to vary among species (Licht 1966). Body temperatures maintained during activity by congeneric species of lizards are quite similar even in widely different habitats, whereas less closely related species of lizards have very different *T*_b_ even when sympatric (Bogert 1949, 1981; Brattstrom 1965). Given that *T*_b_ during activity does not vary much in different habitats irrespective of the local temperatures, one would not expect much variation in the selected temperatures (*T*_p_) within a population or species. However, estimates about phenotypic variation within a population or species with respect to *T*_p_ or *T*_b_ are rarely available. Also, very little is known about heritability or the physiological bases of temperature preference. In the context of current climate change (Kearney et al. 2009; Sinervo et al. 2010; Huey et al. 2012) such knowledge about presumed optimal temperature or *T*_p_ of a population is essential to determine whether rapid climate change is indeed putting selection pressure on the population in question. If climate change is acting as a selection pressure, evolving higher temperature preference (*T*_p_) or higher activity temperature range could be one of the ways populations adapt to the higher temperatures. However, estimates of the phenotypic variation available in the population with respect to temperature related traits, as well as estimates of the heritability of the traits, are essential to predict how the population will respond to changing climate.

The side-blotched lizard *U. stansburiana* (Family Phrynosomatidae) is an annual species that exhibits orange, blue, and yellow alternative throat color morphs in both males and females (Sinervo and Zamudio 2001). Based on field pedigrees (Sinervo and Zamudio 2001), controlled laboratory crosses (Sinervo et al. 2001) and gene mapping studies (Sinervo et al. 2006), throat color and associated life strategies appear to be controlled by a single autosomal locus with three color alleles (o, b, y), hereafter, the “OBY” locus. These codominant alleles give rise to 6 genotypes (oo, bo, yo, bb, by, yy) in both male and female *U. stansburiana* (Sinervo et al. 2006). Previously, Sinervo et al. (2000a, 2001, 2006) have reported different reproductive strategies in *Uta* female throat color morphs. The blue and yellow female morphs (yy, by, bb) lay small clutches of large eggs while orange female morphs (oo, bo, yo) lay large clutches of smaller eggs. A suite of other traits such as phenotypic plasticity in reproduction (Comendant et al. 2003), immunocompetence (Svensson et al. 2001), and corticosterone response to the local social environment (Comendant et al. 2003) are correlated with throat color. This genetically based throat color polymorphism and its correlation with life history and behavioral traits makes this species a valuable system in which to study the effects of increasing local temperatures. The co-occurrence of multiple different trait-value combinations may increase the evolutionary potential of the population and the chances of persistence in the face of changing climatic conditions (Forsman et al. 2008). For instance, different throat color morphs may seek out microhabitats that differ in their thermal properties, be active at different time periods to maintain favorable body temperatures, or may respond differently to increasing temperature due to differences in *T*_p_. Indeed, orange female morphs tend to aggregate where temperatures are consistently high and defend their rock areas more aggressively compared to yellow females (Comendant et al. 2003; Calsbeek and Sinervo 2007). Thermal quality of the females' habitat has significant effect on egg lay date, incubation time, and hatchling survival (Calsbeek and Sinervo 2007). We therefore hypothesize that the throat color alleles might have an indirect effect on thermo-regulatory behavior and/or selected body temperature in *U. stansburiana*.

In this study, we address the following questions using the side-blotched lizard (*U.stansburiana*) as a study system – (1) Does a gravid female lizard's body temperature influence hatchling survival? To answer this question, in 2007, we monitored the body temperatures of gravid females every day in the laboratory until they laid eggs and estimated the hatchling survival to the next breeding season under field conditions. (2) How much phenotypic variation exists in a *U. stansburiana* population with respect to *T*_p_? In 2011, we measured body temperature of about 400 lizards from a single population in a laboratory thermal gradient to address this question. (3) To what extent do sex, reproductive status, and life history stage contribute to this phenotypic variation? (4) Do throat color alleles (OBY) influence thermoregulatory behavior or selected temperature (*T*_p_)? The contributions of sex, reproductive status, and throat color genotypes to phenotypic variation in *T*_p_ were inferred from the 2011 dataset. (5) Is the phenotypic variation in *T*_p_ within a *U. stansburiana* population due to additive genetic variation and/or maternal effects? A laboratory breeding experiment complimented by *T*_p_ measurements of the dams, sires, and hatchlings allowed us to estimate relative contributions of additive genetic variation and/or maternal effects to phenotypic variation in *T*_p_.

## Materials and Methods

### Field site and lizard capture

All protocols involving live animals were approved by the University of California, Santa Cruz Institutional Animal Care and Use Committee (IACUC, Office Code: Sineb1203). All lizards were caught by noosing from a long-term field study site at Los Baños Grandes, CA (37 03′ 29.98″ N, 120 50′ 56.00″ W, 35 m) during spring 2007 and 2011. The habitat mostly consists of sandstone rock outcrops and rock piles on grassy hillsides. Throat colors were scored immediately after capture, according to a protocol described earlier (Sinervo et al. 2001, 2006).

### Does a gravid female lizard's body temperature influence hatchling survival?

In 2007, gravid females were caught by noosing from the field. They were then brought to the laboratory, weight and snout-vent length (SVL) were measured, and unique toe clips were given for individual identification. They were housed individually in terraria with a substrate of moist peat moss and sand, with a rock for basking. A heat lamp with a 40 W light bulb (one lamp for every four terraria) created thermal gradient of 18–37°C within each terrarium. Heat lamps were on from 0800 to 1900 h each day, and lizards were also exposed to the natural photoperiod through glass ceiling of the greenhouse where they were kept. Body temperature was measured for gravid females (*n* = 302) using a BAT-12 electronic thermometer (Physitemp Instruments, Clifton, NJ) with a T-type thermocouple flexible implantable probe (Harvard Apparatus, Holliston, MA) coated with petroleum jelly. The probe was inserted approximately 5 mm into the lizards' cloaca. The *T*_b_ measurements were taken throughout the day at various times (but not more than once a day for each individual). The eggs laid by the gravid females were incubated in individual egg cups at 28°C following a protocol described in Sinervo and Doughty (1996). The hatchlings born in lab were weighed, given unique toe clips for individual identification, and released within 4 days of hatching to the field exactly at the spot where their mother was captured. In spring 2008, the surviving hatchlings were recaptured to estimate the hatchling survival rate to maturity. Repeated visits and mark-recapture sampling of the sites in previsous years allowed 98% recovery of surviving hatchlings were (Sinervo et al. 2006).

### How much phenotypic variation exists in the population with respect to selected temperature (*T*_p_)?

Selected temperature (*T*_p_) refers to the arithmetic average of body temperatures of animals in a laboratory thermal gradient or equivalent conditions that would permit an animal to extend its body temperature above and below the activity temperature range (Pough and Gans 1982; Table 1). In 2011, we measured *T*_p_ in a laboratory thermal gradient using a modification of the protocol described in Sartorius et al. (2002) (Paranjpe et al. 2012). The experimental set up consisted of 24 parallel tracks made out of smoothened particle boards. Each running track was 91.5 cm × 15 cm × 38 cm (length × width × height). A thermal gradient was created in each running track using a heating lamp (100 W full spectrum) at one end. Aluminum foil was used to cover the hot end of the track and around the heating lamp so as to concentrate the heat in the track and to minimize loss of heat to surroundings during the experiment. In this set up, we achieved a thermal gradient of 48–25°C in the running tracks. The temperature gradient in the tracks was continuously monitored during the experiment using small-digital thermometers. For measuring the body temperature of individual lizards we inserted an epoxy coated (1 mm diameter, final probe size) ultra-thin thermocouple probe (Omega T-type junction probes) into cloaca of the lizard and taped the probe around the tail to keep it in place. The other end of the probe was plugged into a data-logger (Eltek Squirrel 1035 series), which automatically records body temperature of 24 individuals simultaneously at 1 min intervals. The lizards attached with thermocouple probes were placed in the thermal gradient and allowed to acclimatize for 10–15 min after which their *T*_b_ was recorded continuously for 2 h. The body temperatures of 391 adults (303 females, 88 males) and 73 (33 females and 40 males) hatchlings were recorded in 2011 using this method. *T*_b_ of adults was measured in the thermal gradient within a week after their field capture.

**Table 1:** Frequently used terms related to thermoregulation and their definitions (adopted from Pough and Gans 1982)

Body temperature (*T*_b_)	It is used in general sense to indicate an approximate internal temperature. In most studies on ectotherm thermoregulation, *T*_b_ refers to the average field body temperature.
Selected (preferred) temperature (*T*_p_)	The arithmetic average of the body temperature measured from animals in a laboratory thermal gradient. This assumes a normal distribution of body temperatures in the population. If the distribution is not normal, other measures such as median can be used to calculate *T*_p_ of the population as well as individuals.
Selected temperature range	The range of body temperatures maintained by an ectotherm in a laboratory temperature gradient providing conditions that would permit an animal to extend its body temperature above and below the activity temperature range.

After recording body temperature in a thermal gradient gravid females were housed individually in small bins with moist substrate until they laid eggs. *T*_p_ of some of these females (*n* = 22) was recorded again in the thermal gradient as described earlier within a week after laying eggs. After recording the postlay *T*_p_ they were released to the exact spot in the field where they were captured. Eggs were incubated individually in vermiculite substrate at 28°C until hatching, according to a protocol described earlier (Sinervo and Doughty 1996). This temperature maximizes survival to maturity (Calsbeek and Sinervo 2007). The hatchlings were weighed and given unique toe clips for future identification. They were released back in the field within 4 days of hatching exactly at the spot where their mother was captured.

### Is the phenotypic variation in *T*_p_ within a *Uta stansburiana* population due to additive genetic variation and/or maternal effects?

To estimate the contribution of additive genetic variation and/or maternal effects to phenotypic variation in *T*_p_ we set up a controlled laboratory breeding experiment in 2011. The protocol for the laboratory breeding experiment was similar to one described earlier (Sinervo et al. 2001; Lancaster et al. 2007). Briefly, we caught adults as soon as they came out of hibernation at Los Banos, CA during early spring of 2011 and used a nested half-sib design for the breeding experiment. *Uta stansburiana* is an annual species and most individuals at Los Banos field site do not survive beyond one breeding season. All females caught for the breeding experiment were born during early June through end of August of the previous year (i.e., 2010). Female follicular development was assessed by abdominal palpation after field capture (Sinervo and Doughty 1996), and only females in the early stages of follicular development (before they had become receptive to mating in that breeding season) were used in the breeding experiment. Previous mating studies have found that sperm is not stored across seasons (B. Sinervo, unpubl. data). Therefore, we can be reasonably sure that the females involved in breeding experiment were virgins and had not stored sperm from previous year. We first measured *T*_p_ of dams and sires before housing them together in the breeding bin. A total of 40 field caught males (sires) were each mated to 2–3 field caught females. Thus, breeding groups consisted of 2–3 females and a single male, all of known throat color genotypes, housed in bins (58.4 cm L × 41.2 cm W × 31.4 cm H) with sand substrate. The females housed in the breeding bins were size matched to minimize the potential effects of body size on female-female competition and male mate choice. Spot lights of 75 W were used to create a thermal gradient of 20–40°C in the breeding bins. The lizards were fed crickets ad libitum and had free access to rock piles which could be used as refuges. We set up crosses which represented combinations of all six throat color genotypes (oo, ob, yo, bb, by, yy). Every other day females were checked for follicular development by abdominal palpation. Once females became gravid, they were moved to smaller bins with moist substrate and housed individually until they laid eggs. Eggs were incubated individually in vermiculite substrate at 28°C until hatching according to a protocol described earlier (Sinervo and Doughty 1996). The hatchlings were weighed and given unique toe clips for future identification. They were housed individually in bins with soil and sand substrate under 40 W full spectrum spot lights as well as UVB tube lights (ZooMed Repti-Sun UVB 5.0). Twice a day, they were fed ad libitum pinhead crickets (*Acheta domestica*) dusted with calcium powder (without D3) and reptile vitamins (both from Zoo Med). Out of 175 hatchlings that were born from the breeding experiment, 76 survived to the age of 2 months (43% survival) when we could measure their *T*_p_. Hatchling survival rate at the field site for this species ranges from 10% to 25% (B. Sinervo, unpubl. data). Therefore, it is reasonable to assume that we had hatchling survival rates better than field survival rates. The proportion of hatchlings surviving in the lab breeding experiment was not influenced by their mother's *T*_p_ and/or genotype.We measured *T*_p_ of these lab-bred hatchlings when they were about 2-months-old, using a protocol similar to that described above for adult lizards. Considering the very small size of hatchlings (0.6–2.0 g) at 2 months of age, body temperature of hatchlings was measured by attaching the ultra-thin thermocouple probe to each hatchling's belly using cyanoacrylic cement and a small patch of tape to insulate the probe, instead of the cloacal probe which would be too large for hatchlings. The *T*_b_ values measured using cloacal probe and belly probe might be different, hence we compared the measurements by using both methods on 24 adult lizards (12 males and 12 females). For the calibration of two methods the lizards were fitted with two probes simultaneously, one in the cloaca and another on the belly. Their *T*_b_ was, thus, monitored using both methods at the same time on same set of lizards. Measuring *T*_b_ for both parents and hatchlings at the age of 2 months enabled us to estimate the contributions of additive genetic variation and maternal effects to phenotypic variation in *T*_p_.

## Data analyses

All statistical analysis was done using JMP 9.0 (SAS Institute 2010), unless otherwise noted. For the 2007 data, we calculated average *T*_b_ for each individual gravid female based on multiple measurements taken at various time points during the day. This value for each female is referred to as selected temperature (*T*_p_) henceforth. Effects of mother's *T*_p_, mother's throat color genotype, hatchling sex, hatchling mass, and the interaction between mother's *T*_p_ × mother's throat color genotype on hatchling survival to next year were tested using nominal logistic regression, with surviving hatchlings given a score of 1 and dead hatchlings given a score of 0. Factors found to be not significant were eliminated in a step-wise procedure. The final model included mother's *T*_p_, mother's throat color genotype, and the interaction of mother's *T*_p_ by mother's throat color genotype. For 2011 data, we calculated the average and median *T*_b_ values for individual adult lizards from the 2-h continuous body temperature recordings under the laboratory thermal gradient. We also calculated the variance of *T*_b_ values of individuals to estimate phenotypic variation (*V*_p_) available in the breeding adults of the Los Banos population. The body temperature data were checked for normality of distribution using the Shapiro–Wilk *W* test. Selected or preferred temperature (*T*_p_) was calculated as the median of individual body temperature values (obtained from recordings in laboratory thermal gradient), while interquartile range (IQR, 25–75% portion) of the distribution of individual *T*_b_ values represented the “selected temperature range” (Dewitt and Friedman 1979; Pough and Gans 1982; Christian and Weavers 1996).

We assigned throat color scores of 0, 1, or 2 for lizards on orange, blue, and yellow axes depending on the description of OBY genotype made at the time of field capture (Sinervo et al. 2001, 2006). For example, on the orange axis, lizards with no visible orange coloration (bb, by, yy genotypes) get a 0 score, individuals expressing orange and another color (bo, yo genotypes) get a score of 1, and individuals with pure orange throats (oo) get a score of 2. On the blue axis, absence of b allele (oo, oy, yy) get a 0 score, heterozygotes of b (by, bo) get score 1 while a blue homozygote (bb) gets score 2. Similarly, on y scale, absence of y allele (oo, bo, bb) get a 0 score, heterozygotes of y (yo, by) get score 1 while a yellow homozygote gets score 2.

Females were categorized into three groups depending on their reproductive status – (i) gravid females, (ii) those which had recently laid eggs (postlay females), and (iii) females of reproductive age that did not become gravid (nonbreeding females). There were no such categories for males.

The distribution of body temperature in the *U. stansburiana* population was not normal (Shapiro–Wilk *W* test, *W* = 0.96, *P* < 0.001). We tried various analysis using both average and median values (calculated from body temperature records of each individual) and found that using either average or median *T*_b_ does not change the results qualitatively. Hence, we considered median *T*_b_ values of individual lizards for further analyses, unless otherwise mentioned. The residuals of the models used were normally distributed when we used median as our response variable (Shapiro–Wilk *W* test for goodness of fit, *P* = 0.2769). Very large sample size (*n* = 198) in this analysis allowed us to assume asymptotic normality. Henceforth, the selected temperature (*T*_p_) will refer to median of individual body temperatures measured in laboratory thermal gradient. We analyzed whether starting time of the trial, sex, and throat color score had any influence on *T*_p_ values of individual lizards by fitting a generalized linear model. For this analysis, we considered only males and nongravid females (nonbreeding + postlay females). Further, data for females only was considered to test the effects of reproductive status, throat color score on Y axis (fixed factors), and starting time of trial on *T*_p_ using a generalized linear model. Similar analyses were done using O and B throat color scores. As the results did not change qualitatively, we have used throat color scores on Y axis in similar analyses, unless otherwise mentioned. We also checked whether maternal *T*_p_ had an effect on egg survival using linear regression.

Hatchling body temperature data were checked for normality of distribution (Shapiro–Wilk *W* test) as well as influence of starting time of trial and sex on *T*_p_ by fitting a linear model. Heritability of *T*_p_ was estimated by regressing hatchling *T*_p_ on dam and sire's *T*_p_ separately. For estimating heritability, we first pooled body temperature data from all hatchlings born to same mother and calculated average *T*_p_ of siblings, which gave us a more accurate estimate of heritability compared with taking *T*_p_ of each hatchling without considering sib-ships. We then estimated heritability of *T*_p_ by regressing average *T*_p_ values of sib-ships (*n* = 30) on the average *T*_p_'s of dam and sire separately. For the heritability analysis, we used dam's *T*_p_ when she was done laying eggs (postlay *T*_p_), thus avoiding the effect of the temporary change in *T*_p_ during gravid condition which may lead to an erroneous heritability estimate. Even though we had 40 sires and more than 120 dams at the beginning of the breeding experiment, *T*_p_ data for only those sires and dams that had hatchlings surviving up to the age of 2 months could be considered for heritability analysis.

To estimate the extent of maternal effects on *T*_p_ of hatchlings, we used statistical methods suggested by Fry (2004). For this analysis, a traditional variance component model (Sokal and Rohlf 1981) can be applied to body temperature data:


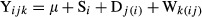


Here, Y_*ijk*_ is an observation, μ is the population mean, S_*i*_ is the random effect of the *i*th sire, D_*j*(*i*)_ is the random effect of *j*th dam within the *i*th sire group (because it is a nested half-sib design), and W_*k*(*ij*)_ is the random effect of the *k*th hatchling within the *ij*th family. The three random effects are assumed to be independent and normally distributed with means zero and variances 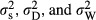, respectively. (Note that “D” here stands for dam, not dominance.) These are “observational components” of variance because they can be calculated from observed data. To estimate the additive genetic variance (*V*_A_), the common environment or maternal-effect variance (*V*_M_), and the within family environmental variance (*V*_E_), the following equations can be used (Falconer and Mackay 1996):


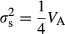



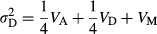



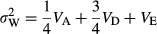


Assuming dominance variance, *V*_D_, equals zero, we can solve for the causal components:


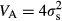



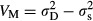



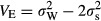


PROC MIXED program in SAS (Fry 2004) was used to provide Restricted Maximum likelihood (REML) estimates of the observational components of variance (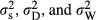). These estimates were used to estimate the causal components (*V*_A_, *V*_M_, and *V*_E_) using the equations above.

## Results

### Mother's *T*_p_ influences hatchling survival in the field

In 2007, mother's *T*_p_ (obtained from measurements in terraria), mother's throat color genotype, and the interaction between mother's *T*_p_ by mother's throat color genotype had significant effects on field hatchling survival to the next year (Table 2). Hatchling survival was not affected by weight at birth (Table 2).

**Table 2:** Nominal logistic regression for factors influencing hatchling survival to next breeding season in *U. stansburiana* population

Source	df	L-R Chi square	*P*>Chi square
Dam *T*_b_	1	5.20375	0.0225*
Dam genotype	5	12.11031	0.0333*
Dam *T*_b_ × Dam genotype	1	11.8007	0.0376*
Hatchling mass	1	1.48537	0.2229
DamID (HatchID)	1	0.028442	0.8661

* *P* < 0.05.

### Phenotypic variation in *T*_p_ and factors contributing to the variation

In 2011, body temperature of 303 females and 88 males was recorded in a laboratory thermal gradient. For all further calculations and analyses mentioned in the text, we used data collected only in 2011 using the laboratory thermal gradient and the breeding experiment. Therefore, all *T*_p_ values mentioned henceforth are those calculated using 2011 data. Average *T*_b_ of the *U. stansburiana* population was 37.36 ± 0.086°C (mean ± SE), while median *T*_b_ was 37.81°C. The estimate of phenotypic variance (*V*_p_) of the population with respect to *T*_b_ was 2.92. Selected or preferred temperature (*T*_p_) range for the population, calculated as interquartile range of the average *T*_b_ distribution was 36.49–38.56°C.

**Figure 1:**
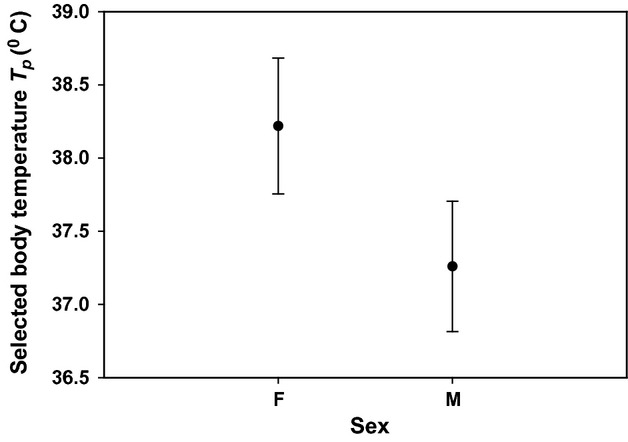
*T*_p_ for nonbreeding females (*n* = 111) was significantly greater than *T*_p_ of males (*n* = 87). Error bars indicate mean ± SE.

Significance of factors contributing to the phenotypic variation in *T*_p_ values was tested using generalized linear models. Color score on Y scale and starting time of the trial did not have a significant effect on *T*_p_ of males and nonbreeding females (*n* = 196, Table 3). Analyses using the O and B scales gave similar results. Sex significantly influenced *T*_p_ (Table 3). Post hoc comparisons using Student's *t*-test revealed that *T*_p_ for nonbreeding females (*n* = 109) was significantly greater than *T*_p_ of males (*t* ratio = −5.75204, *P* < 0.0001) (*n* = 87, Fig. 1).

**Table 3:** Generalized linear model for factors influencing *T*_p_ of adults (males and females) from *U. stansburiana* population

Source	df	*F* ratio	*P*>*F*
Sex	1	33.0859	<0.001*
Color score (Y scale)	4	0.3385	0.8518
Starting time of trial	1	1.7521	0.1872

* *P* < 0.05.

**Figure 2:**
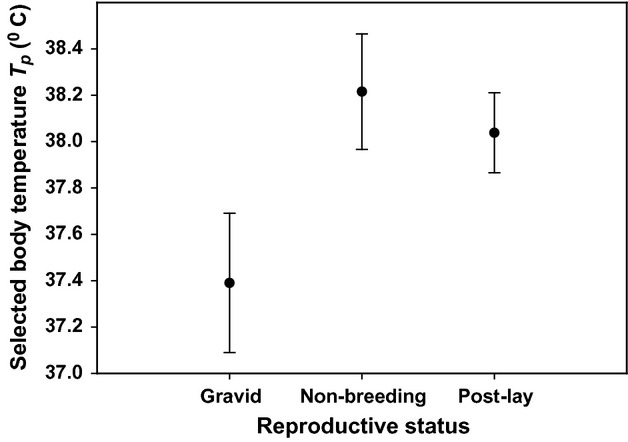
Gravid females had significantly low (about 1.0°C lower) *T*_p_ compared to postlay and nonbreeding females. Error bars indicate mean ± SE.

Starting time of trial and throat color score on Y scale had no significant effect on the *T*_p_ of females (*n* = 259) when considered separately from males, while reproductive status had a significant effect on *T*_p_ of females (Table 4). Post hoc comparisons using student's *t*-test revealed that gravid females had about 1.0°C lower *T*_p_ compared with postlay and nonbreeding females (*t* ratio = 3.20591, *P* = 0.0015; Fig. 2). Therefore, we measured body temperature for a set of 22 females when they were gravid and within a week after they laid eggs. Paired comparisons of *T*_p_ values before and after laying eggs using *t* test showed that *T*_p_ was higher after laying eggs than before by 2.92 ± 0.55°C (mean ± SE) (paired *t*-test, *P* < 0.001). However, there was no significant correlation between *T*_p_ of females when they were gravid and the postlay *T*_p_ (Ranked correlation, *R*^2^ = 0.0085, *P* = 0.6828). *T*_p_ of gravid females did not influence the proportion of eggs surviving to become hatchlings (*F* ratio = 0.0926, *P* = 0.7629). We measured hatchling survival (born in 2011) to next breeding season (2012) and found that there was very low hatchling survival in 2012 (8 out of 200 hatchlings released in 2011 survived to 2012, compared to 50 out of 484 in 2007–2008). Therefore, we did not have enough power to detect the effect of mother's *T*_p_ on hatchling survival for the 2011–2012 data and could not compare it to 2007–2008 results.

**Table 4:** Generalized linear model for factors influencing *T*_p_ of adult females

Source	df	*F* ratio	*P*>*F*
Reproductive status†	2	5.2295	0.006*
Color score Y scale	4	2.0151	0.093
Start time of trial	1	0.704	0.791

† Females were considered in three categories: gravid, postlay, and nonbreeding females. For detailed description of categories please refer to the text.

* *P < 0.05.

**Figure 3:**
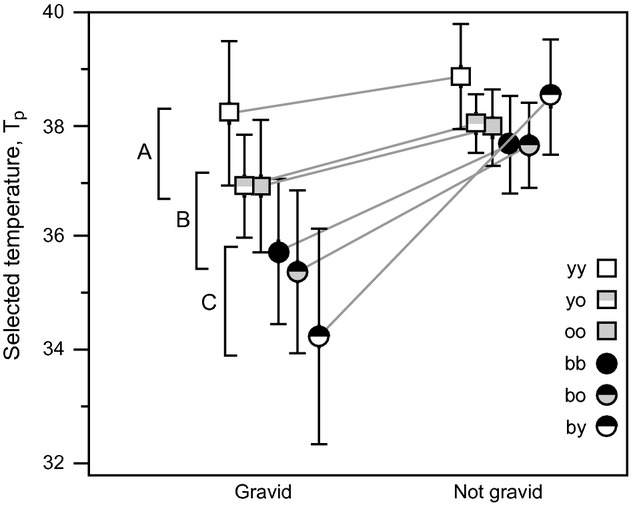
Effect of throat color genotype × reproductive status interaction on *T*_p_ of females. Error bars indicate mean ± SE. For this analysis, females were grouped into gravid and nongravid categories (by pooling postlay and nonbreeding females into nongravid category). YY females, overall, have higher *T*_p_ compared with other genotypes. Y and O females tend to prefer higher temperatures when gravid compared with BB females. The letters A, B, C indicate genotypes that were significantly different from other genotypes.

As postlay and nonbreeding females have similar *T*_p_ (Fig. 2), we pooled them in one category called nongravid females. We then compared the *T*_p_ of females including a factor for gravid versus nongravid females, covariates for Y scale and O scale, and interaction terms for gravid versus nongravid by Y scale and gravid versus nongravid by O scale. The gravid versus nongravid condition of females, their Y color score and the interaction between gravid versus nongravid condition × O scale had significant effect on median *T*_b_ (Table 5). The interaction between gravid versus nongravid condition and Y scale was not significant (Table 5). Thus, females with O alleles (oo, yo genotypes) had higher *T*_p_ when gravid, compared to the females with B alleles in general (bb, by, bo genotypes). Furthermore, females with two Y alleles had higher *T*_p_ compared to females with 0 or 1 Y allele (Fig. 3).

**Table 5:** Effects of reproductive status and throat color genotype on *T*_p_ of females

Source	df	*F* ratio	*P*>*F*
Gravid vs. Nongravid	1	25.0802	<0.0001*
Color score (Y scale)	1	8.1128	0.0047*
Color score (O scale)	1	2.0256	0.1558
Y scale × Gravid vs. Nongravid	1	3.2002	0.0747
O scale × Gravid vs. Nongravid	1	4.8078	0.0292*

* *P* < 0.05.

### Temperature preference in hatchlings and the heritability of *T*_p_

**Figure 4:**
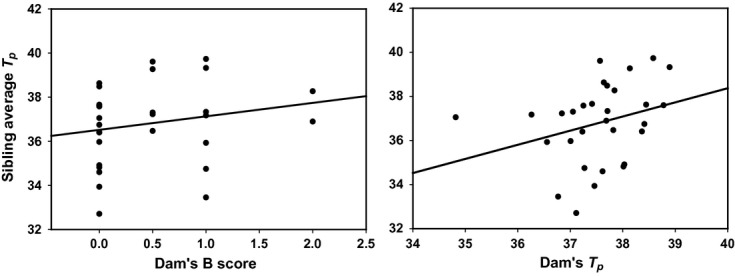
Dam's *T*_p_ and throat color score on B axis had significant influence on hatchlings' *T*_p_.

**Figure 5:**
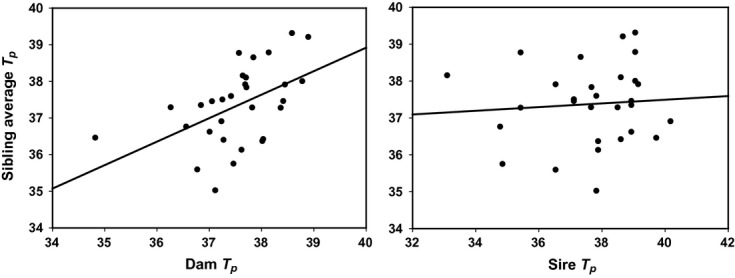
Regression of sib-average *T*_p_ (*n* = 30 sibships) on dam's *T*_p_ (left panel) and on sire's *T*_p_ (right panel). *T*_p_ of dam, but not sire's *T*_p_, had significant influence on *T*_p_ of hatchlings.

Comparison of *T*_b_ measured using cloacal probes and belly probes showed that there was a significant correlation between the two readings (*R*^2^ = 0.69, *P* < 0.0001) and body temperature recorded using belly probe was on an average 0.55°C higher compared that recorded using cloacal probe (Wilcoxon signed rank test, *P* = 0.0191). This value was used to calibrate the adult *T*_b_ to hatchling *T*_b_ values before further analysis. Average *T*_b_ value of hatchlings (*n* = 73) was 36.70 ± 0.19°C (mean ± SE). Selected temperature range of hatchlings, calculated using interquartile range, was 35.72–37.86. The *T*_p_ values of hatchlings were not influenced by their sex or their sire's OBY genotype, so we excluded these two factors from further analyses. However, dam's *T*_p_ and OBY genotype (B scale) both had significant effects on sib-average *T*_p_ (Table 6, Fig. 4). Dams with higher color score on B scale produced hatchlings with higher *T*_p_ (Fig. 4, left panel), while dam's *T*_p_ had positive influence on hatchling *T*_p_ (Fig. 4, right panel). Heritability through the sire was not significant (*h*^*2*^ = 0.08, *P* = 0.7343), however, the slope of the regression line on dam's *T*_p_ was 0.64, which yielded a heritability estimate of *h*^*2*^ = 1.28 ± 0.93 (Fig. 5). The analyses using SAS gave us estimates of additive genetic variance through the sire (*V*_A_ = 0), variance due to maternal effects (*V*_M_ = 0.62 ± 0.29) and within-family variance (*V*_E_ = 0.5 ± 0.7).

**Table 6:** Linear model for factors influencing calibrated sib-average *T*_p_ of hatchlings

Source	df	*F* ratio	*P*>*F*
Dam average *T*_b_	1	8.9364	0.0062*
Sire average *T*_b_	1	0.0476	0.8291
Dam B score	1	4.2518	0.0497*

* *P* < 0.05.

## Discussion

We estimated the phenotypic variation present in one population of *U. stansburiana* with respect to *T*_b_ using a large sample size (*n* = 391, i.e., 303 females and 88 males) as well as the selected temperature range of the population (36.49–38.56°C). These estimates are essential to predict the evolutionary response of the population to local warming trends. Such estimates are not available for other populations of *U. stansburiana* for comparison as most previous studies have used small sample sizes to estimate average population *T*_b_ and *T*_p_ range (Parker and Pianka 1975; Waldschmidt and Tracy 1983) and/or have not reported variance. Average and median *T*_b_ values as well as *T*_p_ range reported here (37.36 ± 0.086°C, 37.81°C, and 36.49–38.56°C, respectively) are higher than previously reported *T*_b_ and *T*_p_ values for the species using similar methods (average = 35.5°C, median = 36.2°C, *T*_p_ range = 32.9–38.3°C by Sartorius et al. 2002). *T*_p_ range for *U. stansburiana* population in our study seems to be narrower and slightly higher compared to the New-Mexico population in the earlier study. The New Mexico (NM) study site mentioned in Sartorius et al. is a xeric shrubland with sandy dunes. Our study site is a cattle ranch with grassy slopes and rock outcrops. NM site probably has greater variation in daily temperature due to inland drier habitat compared to CA study site that might explain the broader selected temperature range in the NM population. However, higher average and median *T*_b_ values in our study population compared to NM population are unexpected and could reflect population level differences that are not dependent on habitat type.

Various factors can contribute to variation in body temperature, and we investigated effects of time of day (starting time of trial), sex, reproductive status, and throat color genotype of the individual. Analysis of our data revealed significant effects of sex and reproductive status on *T*_p_. Female lizards in our study had about 1° higher *T*_p_ than males as measured in laboratory thermal gradient. Earlier studies on lizards have reported that males may select similar (Lailvaux et al. 2003; Ibargüengoytía 2005), higher (Pentecost 1974) or lower *T*_b_ compared to females (Sievert and Hutchison 1989). In our field observations, we notice that males become active and start basking slightly earlier during the day compared to females (B. Sinervo, pers. obs.). Also, overall, *U*. *stansburiana* males come out of winter hibernation before females (B. Sinervo, unpubl. data). Lower *T*_p_ of males might explain why they become active earlier during the breeding season as well as during the day when the air temperatures are lower. Similar sex differences in thermal sensitivity were reported in *A. carolinensis* (Licht 1973a). Such differences might ensure that males are sexually competent and active before females start investing resources in ovarian and egg development (Licht 1973a). However, gender differences in body temperature such as those observed in our study are the exception, not the rule, especially in case of desert lizards (Huey and Pianka 2007). A review of previous reports indicates that mean body temperatures of males and females differ by less than 1°C in 80.4% of lizard species (Huey and Pianka 2007), thus, the gender differences observed here may be biologically interesting.

Interestingly, reproductive status also changed *T*_p_ of females such that the gravid females had about 1.5°C lower *T*_p_ compared to postlay and nonbreeding females. To confirm whether reproductive status really changes *T*_p_ of females, we monitored the body temperature of a set of 25 females when they were ready to lay eggs and within 7–10 days after they laid eggs (postlay). After laying eggs, the females' *T*_p_ increased by 2.92°C on average compared to when they were gravid. Therefore, lower *T*_p_ in gravid females seems to be a temporary state. The difference is probably not due to relative inactivity of gravid females, as the females were moving between warmer and colder spots in the thermal gradient (D. Paranjpe, pers. obs.). One possible reason for the lower *T*_p_ of gravid females could be that temperatures higher than a certain threshold may harm developing embryos (Beuchat 1988). Neither average nor median *T*_b_ of gravid females influenced egg-to-hatchling survival in our population. However, in our experimental protocol as well as under field conditions, the egg-to-hatchling survival is measured after the eggs have been laid, that is, when the egg temperature is no longer dependent on mother's body temperature. Therefore, it is possible that higher temperatures, if experienced by gravid females, may be harmful for the developing eggs and are avoided by gravid females when they can choose appropriate temperature for activity. Alternatively, the deleterious effects of higher temperatures might manifest themselves in hatchling survival to adulthood. Indeed, analysis of 2007 data revealed that body temperature of gravid females did influence hatchling survival to the next year in the Los Banos population (Table 2). Females with genotype “by” had lowest *T*_b_ when gravid (Fig. 4) and had the highest number of surviving hatchlings the next year. Therefore, mother's *T*_p_, when gravid, seems to be important for survival of the hatchlings. Similar to our results, pregnant females of the viviparous lizard *Sceloporus jarrovi* gave birth to significantly more abnormal or dead offspring when maintained at constant high temperatures (Beuchat 1988). However, field body temperatures of gravid females of oviparous gekkonid lizards were higher than those of nonreproductive individuals (Werner 1990). Reproductive status can change *T*_b_ in snakes (Peterson et al. 1993) and viviparous squamates (Shine 1980). Thus, from various studies it appears that reproductive status can change temperature preference in either direction. The fitness consequences of the temperature change may need further investigation in those cases.

O female genotypes (oo, yo), in general, had higher *T*_p_ when gravid, relative to B female genotypes (bb, by, bo) (Fig. 3). Previously, Sinervo et al. (2000a) have reported different reproductive strategies in *U. stansburiana* female throat color morphs and other correlated traits such as phenotypic plasticity in reproduction (Comendant et al. 2003), immuno-competence (Svensson et al. 2001), and corticosterone response to the local social environment (Comendant et al. 2003). Likewise, the elevated *T*_p_ of orange females when gravid compared to blue females (Fig. 3) indicates yet another component of body temperature plasticity important for reproductive strategies of females. Our results raise an interesting possibility that different genotypes may choose egg-laying sites that differ in their thermal properties and thereby influence the rate of embryo development and/or hatchling thermal preference through developmental acclimation (Angilletta et al. 2006). However, this possibility remains to be tested.

Hatchlings born from the laboratory breeding experiment had a normal distribution of body temperature values, unlike adult lizards. Hatchlings had similar *T*_p_ irrespective of their sex. It is notable that the temperature preferences of male and female hatchlings are not significantly different, while adult females (nonbreeding and postlay) have higher *T*_p_ compared to males. It, therefore, appears that female (or male) *T*_p_ might change during the transition from hatchling to adult stage.

In our laboratory experiment, dam's throat color genotype, but not sire's genotype, influenced hatchling's *T*_p_. Furthermore, regression analysis showed that *T*_p_ is heritable through the mother and not through the sire. Traits such as throat color (Sinervo and Lively 1996; Zamudio and Sinervo 2000), body size (Calsbeek and Sinervo 2004), and immune response (Svensson et al. 2001) are known to be heritable through both males and females in *U. stansburiana,* while egg lay date, egg size (Sinervo and Doughty 1996), and clutch size (Sinervo and McAdam 2008) are heritable through females. Dorsal patterns in *U. stansburiana* are heritable but also modulated by inducible maternal effects (yolk steroid hormones, Lancaster et al. 2010). Previously, Lancaster et al. (2010) showed that females of *U. stansburiana* can induce variable dorsal patterns in progeny via manipulation of estradiol concentrations in egg yolk. Such maternally induced back patterns are associated with different anti-predator strategies and the progeny with certain combinations of back pattern and anti-predator strategy have higher fitness in the wild (Lancaster et al. 2010). Thus, females can resolve differing correlational selection pressures on different progeny by maternal effects. Temperature preference, which is very important for physiology, behavior, and fitness of lizards, seems to be heritable through dams but not through sires. We therefore checked the possibility that maternal effects rather than additive genetic variation accounted for the variation in hatchling *T*_p_. The nested half-sib design of our breeding experiment allowed us to estimate the relative contributions of additive genetic variation and maternal effects to variation in hatchling *T*_p_ (Falconer 1981; Sinervo 1998). Only one previous study has examined the heritability of body temperature in lizards using the species *Sceloporus occidentalis* (Sinervo 1990). The estimates of genetic sources of variation in that study were potentially confounded by maternal effects and dominance variation (Sinervo 1990) as the calculations were based on *T*_b_ values of full-sibling correlations rather than parent-offspring regression (Falconer 1981; Sinervo 1998).

Ours is the first study to report the influence of maternal effects on physiological trait such as temperature preference in progeny using controlled laboratory genetic crosses. Maternal effects were earlier thought to be ‘troublesome source of environmental variation’ confounding the estimates of heritability (Falconer 1981). However, they have now been recognized as a cause of phenotypic variation that may be important for adaptation in heterogeneous environments (Mousseau and Fox 1998). Maternal effects can manifest themselves at various stages of offspring development such as prezygotic, postzygotic–prenatal and postzygotic–postnatal effects (Wade 1998). In our experimental set up, after females laid eggs, all eggs were incubated under similar conditions eliminating potential postzygotic–postnatal maternal effects due to choice of oviposition site or incubation temperatures (Sinervo and Doughty 1996; Du et al. 2010). Standard incubation temperature (28°C) effectively reduced potential variation in growth rates or hatchling performance due to variation in incubation temperature and gave us more confidence that the variation in hatchling *T*_p_ can be attributed to mother's *T*_b_. The lizards involved in the breeding experiment were housed under similar conditions for breeding and females, when gravid, were moved to separate bins and housed under standard conditions. Thus, we minimized the environmental variation experienced by lizards in the laboratory. The influence on hatchling *T*_p_, therefore, seems to be due to prezygotic and/or postzygotic prenatal maternal effects that are not dependent on recent temperatures experienced by females. Fertilized eggs are retained in the females' oviduct for approximately 10 days before laying, as the various egg layers are added to the recently fertilized and yolky egg (Sinervo and DeNardo 1996). This time frame provides ample opportunity for maternal modulation of thermal preference of progeny. However, the precise developmental status of the preoptic area of the hypothalamus, which controls temperature regulation in both ectothermic and endothermic vertebrates (Romanovsky 2007), is not known for embryos laid by *U. stansburiana* females.

Maternal effects could be seen as a special form of phenotypic plasticity – that is, trans-generational phenotypic plasticity (TPP) because such effects provide a mechanism by which mothers influence future offspring development and phenotype in response to current, predictive cues (Mousseau and Fox 1998). However, the ecological or evolutionary consequences of these maternal effects for the hatchlings' fitness must be estimated to understand whether these effects are truly adaptive or merely a physiological inevitability (Marshall and Uller 2007). In our study, temperatures experienced by gravid females influenced hatchling survival (2007–2008 data, Table 2) such that hatchlings born to dams with lower body temperature were more likely to survive through the winter hibernation. In addition to the direct effect on hatchling survival, temperatures experienced by the mother and the mother's genotype (presence or absence of blue alleles) both influenced hatchling's *T*_p_. Our analyses of natural selection on progeny survival to maturity (Table 2) indicate a significant dam *T*_b_ × dam OBY genotype effect, suggestive of strong correlational selection on the maternal effect mechanism discovered in our study. Similar effects have been found for the other complex maternal determination of OBY × dorsal pattern × maternal estrogen in yolk (Lancaster et al. 2007) or for OBY × egg size that in interaction determine progeny body morphology, dorsal patterns, escape behavior that influence survival (Lancaster et al. 2010). We suggest that such maternal effect machinery must entail selection on complex epistatic mechanisms involving multiple genes and multiple fitness optima. For this thermoregulatory maternal effect, the effect of dam's blue allele on hatchling *T*_p_ cannot be due to higher temperature experienced by bb females when gravid, because bb females have low *T*_p_ when gravid compared to yy and oo females (Fig. 3). The significant effect of dam *T*_p_ on progeny *T*_p_, along with the significant effect of dam's blue color alleles on progeny *T*_p_ (Fig. 4), and the corresponding lack of a heritable effect through the sire (Fig. 5) indicate that *T*_p_ is controlled largely by a maternal effect that is associated with dam throat color alleles, and then can persist over generations via the impact of dam *T*_p_ per se. The developmental control of progeny *T*_p_ is, therefore, due to a persistent maternal effect and under selection in nature. In the ecological context, it might mean that the progeny of dams with blue alleles are more likely to survive in hotter environments and/or that dams with blue alleles might choose to lay eggs later in the breeding season compared to other genotypes. Additional data on how the pattern of selection on *T*_p_ changes over the long-term will be required to resolve such issues.

This result has important implications under the current climate change scenario (Parmesan 2006; IPCC 2007) as changes in environmental temperatures can alter body temperature of ectotherms and thus their physiological performance and vulnerability (Kearney et al. 2009; Huey et al. 2012; Moritz et al. 2012). On shorter time-scales and/or under slower climate warming scenarios, maternal effects on hatchlings' *T*_p_ may provide an important buffering mechanism through TPP to mitigate the effects of local warming trends, especially for lizards that do not show long range dispersal. The strength of maternal effects can itself be under selection (Kirkpatrick and Lande 1989) both at maternal and offspring level. Thus, adaptation through maternal effects can occur only if the strength of maternal effects is heritable (Visser 2008). Very little or no effect of additive genetic components on *T*_p_ would, therefore, severely constrain or slow down the evolution of higher *T*_p_ under a rapid climate warming scenario (Sinervo et al. 2010). The mechanisms underlying the persistent maternal effect can only respond rapidly to climate warming if the maternal effects expressed by females with yellow and orange color alleles, which are associated with lower *T*_p_ are reversed or nullified. Otherwise selection for higher *T*_p_ to adapt to a warming climate would necessarily impose correlated selection on the throat color alleles, which are themselves under extremely strong-frequency dependent selection in both males (Sinervo et al. 2006) and females (Sinervo et al. 2000a,b). The strength of the selection in such case will depend on the magnitude and pace of warming.

## Data Archive Plan

All data will be deposited in Merritt Repository Service (http://www.cdlib.org/services/uc3/merritt/) at the University of California Curation Center (UC3) that will manage, archive, and share digital content.
